# Remineralizing and Antimicrobial Effects of a Chitosan–Xylitol Hydrogel on Dental Enamel: An In Vitro Study

**DOI:** 10.3390/gels12060522

**Published:** 2026-06-11

**Authors:** Jesús Antonio Valenciana-Solís, César Gaitán-Fonseca, Luis Aguilera-Galavíz, Gabriel Alejandro Martínez-Castañón, Óscar Cepeda-Argüelles, Verónica Zavala-Alonso, Marco Tulio Bernal-Elías, Carlos Bermúdez-Jiménez

**Affiliations:** 1 Área de Ciencias de la Salud, Universidad Autónoma de Zacatecas “Francisco García Salinas”, Zacatecas 98160, Mexico; 2 Unidad Académica de Odontología, Universidad Autónoma de Zacatecas “Francisco García Salinas”, Zacatecas 98600, Mexico; 3 Facultad de Estomatología, Universidad Autónoma de San Luis Potosí, San Luis Potosí 78290, Mexico

**Keywords:** dental enamel remineralization, hydrogel, chitosan, xylitol, antimicrobial

## Abstract

Dental enamel is a highly mineralized tissue with limited regenerative capacity, making it particularly susceptible to demineralization processes that negatively affect oral health. The development of biocompatible remineralizing materials is essential for advancing preventive and minimally invasive dentistry. This study aimed to evaluate the remineralizing effect of a chitosan–xylitol combination on dental enamel, as well as its antimicrobial activity. It was hypothesized that this combination would promote enamel remineralization. Surface morphology and elemental composition were analyzed using scanning electron microscopy coupled with energy-dispersive X-ray spectroscopy (SEM-EDS). Fourier transform infrared spectroscopy (FTIR) was employed to identify chemical interactions associated with hydrogel formation. Mechanical properties were assessed through Vickers microhardness testing before and after treatment. Additionally, antimicrobial activity was evaluated using the Kirby–Bauer method against microorganisms associated with dental caries. The results demonstrated that the chitosan–xylitol combination promotes enamel remineralization, evidenced by structural changes, increased mineral content, and improved microhardness. A complementary antimicrobial effect was also observed. These findings support the potential of this combination as a promising alternative for preventive and conservative dental treatments.

## 1. Introduction

Dental organs are composed of structures with varying consistencies. The outermost layer is a hard substance called dental enamel, which is extremely hard and therefore considered the hardest substance in the human body. Next is dentin, a mineralized connective tissue that forms the majority of the tooth, beneath the enamel in the crown and beneath the cementum in the root. Dentin also surrounds the dental pulp, which is defined as highly specialized connective tissue residing in the central cavity of the tooth. Structurally, it is highly vascularized and innervated and is composed of a heterogeneous population of fibroblasts, odontoblasts, mesenchymal stem cells, endothelial cells, immune cells, and other elements [1,2,3]. Dental enamel is composed mainly of hydroxyapatite, calcium, phosphate, and hydroxyl ions [4], which explains its high mineral content and its limited regenerative capacity due to the absence of viable cells.

According to the World Health Organization (WHO), dental caries is the most prevalent disease worldwide. It is a condition that affects the dental enamel and is defined as a localized process of multifactorial origin that begins after tooth eruption, causing the softening of the hard tissue of the tooth and progressing to the formation of a cavity [5].

Dental procedures for treating tooth decay involve removing the affected areas and replacing them with metal-based restorative materials, resin matrices, or ceramics [6]. Consequently, patients who receive this type of treatment lose a substantial amount of tooth structure [7].

There is a wide range of products that promise to remineralize enamel, thus preventing the appearance of dental caries or treating initial lesions; these products include fluoride-based products [4], products with casein derivatives [8], products based on amelogenin-derived peptides [9], and even products with synthetic nanohydroxyapatite [10].

Hydrogels based on sodium mannuronate/guluronate (alginate), gelatin, and bioactive calcium silicates/dicalcium phosphate dihydrate derived from cuttlefish bone have emerged as promising biomaterials for mineralized tissue regeneration. Marine-derived sources are particularly attractive due to their natural calcium and phosphate content and bioactive structures that can support apatite formation. These characteristics enable ion release and create a favorable environment for mineral deposition, suggesting their potential application in enamel remineralization strategies [11,12,13].

Several compounds are of interest for preventing tooth enamel demineralization, including fluoride, xylitol, tricalcium phosphate, and chitosan. Some of these compounds have recently garnered attention, such as chitosan, a polysaccharide derived from the deacetylation of chitin, which is obtained from the exoskeleton of crustaceans. Chitosan possesses specific properties that make it interesting in biomedicine, such as biocompatibility, non-toxicity, antimicrobial properties, and the ability to regenerate support hard tissues due to its capacity to sequester calcium and phosphate ions [14,15].

Another compound that has been of interest in biomedical research is xylitol, which is used as a sugar substitute and has been attributed with properties that help prevent tooth decay [16].

Xylitol has been described as having the ability to alter the production of polysaccharides that are essential in the formation of the biofilm that causes dental caries [17]. Xylitol is not fermentable by cariogenic bacteria such as *Streptococcus mutans* and *Streptococcus sanguinis*, which are key contributors to dental caries. Unlike sucrose and other fermentable carbohydrates, xylitol does not serve as a substrate for acid production, thus reducing the risk of enamel demineralization [18]. Furthermore, xylitol inhibits the growth and adhesion of *S. mutans*, likely through multiple mechanisms: it alters bacterial energy metabolism by being absorbed and phosphorylated to xylitol-5-phosphate, which cannot be further metabolized and is excreted at an energetic cost, thereby reducing bacterial growth and virulence [19]. The rationale for combining chitosan and xylitol is based on their potentially complementary mechanisms: chitosan may promote mineral retention and provide antimicrobial and bioadhesive properties, whereas xylitol may reduce acidogenic bacterial activity and support a favorable environment for enamel mineral recovery, suggesting a synergistic effect beyond their individual use [14,15,16,17,18].

The objective of this study is to demonstrate the remineralizing capacity and antimicrobial effect of a chitosan-xylitol based hydrogel.

## 2. Results and Discussion

### 2.1. FTIR Analysis

**Figure 1:**
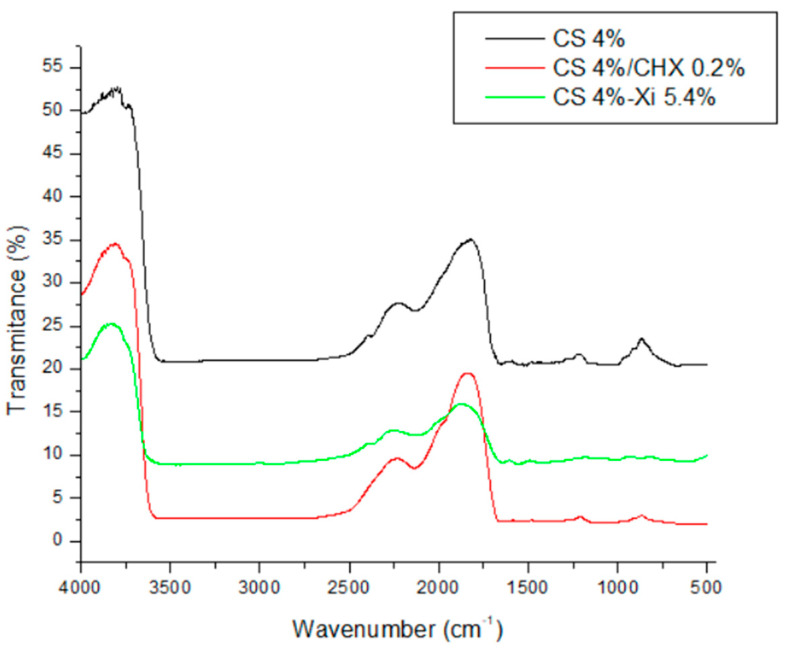
Infrared spectrum of 4% chitosan hydrogel and infrared spectrum of 4% chitosan and 5.4% xylitol hydrogel.

The infrared spectrum of the chitosan hydrogel (CS) shows bands located at 1652 cm^−1^, indicating the presence of amide I bonds (C=O stretching), as well as bands located at 1527 cm^−1^ corresponding to the amine functional group vibrations (Figure 1).

In addition, vibrational bands observed at 1421 cm^−1^, corresponding to O-H bending vibrations, indicate the presence of xylitol in the chistosan-xylitol hydrogel (CS-Xi).

Furthermore, vibrational modes corresponding to amide groups are observed at 1660 cm^−1^, while bands corresponding to amine vibrational modes, characteristic of chitosan, are observed at 1562 cm^−1^.

### 2.2. SEM Analysis

**Figure 2:**
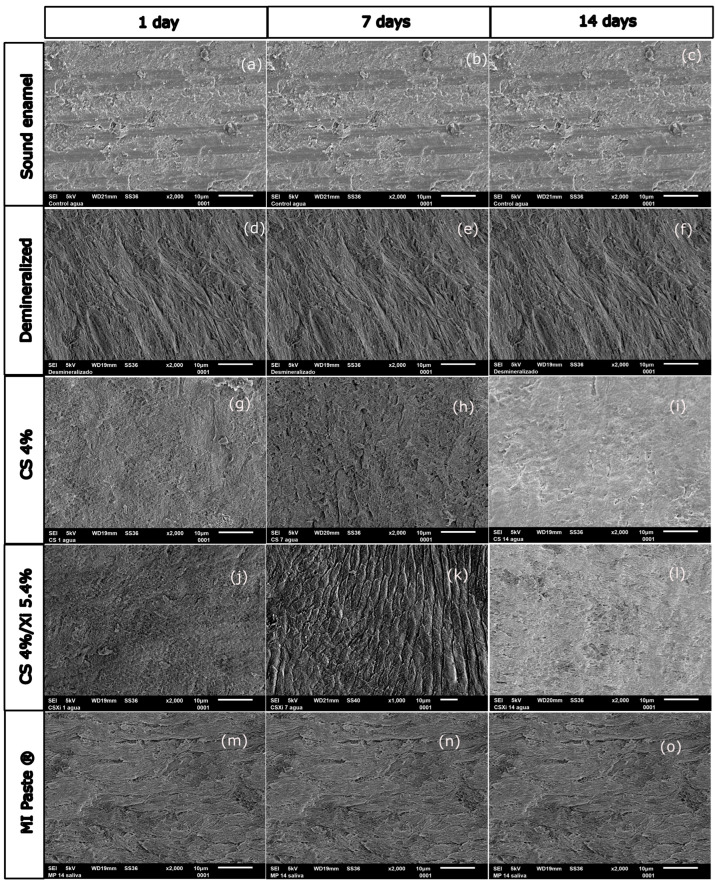
Representative SEM images at 2000× magnification (Scale bars = 10 μm). (**a**) Image of sound enamel. (**b**) Image of sound enamel. (**c**) Images of sound enamel. (**d**) Image of demineralized enamel. (**e**) Image of demineralized enamel. (**f**) Image of demineralized enamel. (**g**) Enamel treated with CS 4% for 1 day. (**h**) Enamel treated with CS 4% for 7 days. (**i**) Enamel treated with CS 4% for 14 days. (**j**) Enamel treated with CS-Xi 5.4% for one day. (**k**) Enamel treated with CS-Xi 5.4% for 7 days. (**l**) Enamel treated with CS-Xi 5.4% for 14 days. (**m**) Enamel treated with MP for one day. (**n**) Enamel treated with MP for 7 days. (**o**) Enamel treated with MP for 14 days.

In the SEM analysis, the control samples (sound enamel) observed at 2000× magnification, revealed a homogeneous, compact, and uniform surface with well-organized enamel prisms. Figure 2a shows the enamel after demineralization with 37% phosphoric acid, exhibiting a loss of prism structure and a rough, poorly compacted surface. Figure 2g shows the enamel treated with CS for 1 day, revealing a surface characterized by loss of structural organization, a rough appearance, and the presence of micropores and cavities distributed across the surface. For the samples treated with CS for 7 days, the enamel surface appeared more homogeneous and compact with a noticeable reduction in surface roughness (Figure 2h).

For the samples treated with CS–Xi (Figure 2j) after 1 day, a homogeneous layer with high surface roughness was observed, along with loss of structural organization and the presence of micropores and cavities distributed across the enamel surface.

For the samples treated with CS–Xi for 7 days (Figure 2k), a regenerated surface layer was observed, characterized by rod-like structures oriented in a similar direction and increased structural density.

For the samples treated with CS–Xi for 14 days (Figure 2l), the enamel surface appeared more compact, with a considerable decrease in demineralized areas. An irregular mineralized layer and partially disorganized rod-like structures were still present, along with isolated areas of micropores and cavities across the enamel surface.

For the control samples treated with MI Paste^®^ (Figure 2m), the enamel surface appeared compact, uniform, and homogeneous, showing continuous surface coverage, rod-like structures, and high structural density. The enamel surface appeared remineralized, with minimal evidence of demineralized areas, micropores, or cavities.

According to the EDX analysis, the Ca/P ratio was 2.6% in sound enamel and 2.7% in demineralized enamel. Following treatment, the Ca/P ratio was 1.9% after 1 day, 1.8% after 7 days, and 2.6% after 14 days (Table 1).

### 2.3. Microhardness Analysis

**Figure 3:**
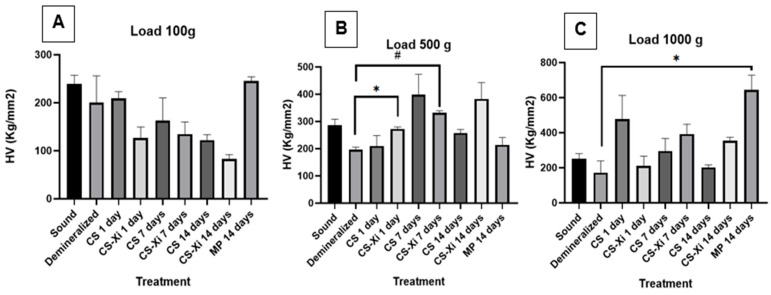
Microhardness analysis. (**A**) 100 gf; (**B**) 500 gf * *p* = 0.0435, # *p* = 0.0115; (**C**) 1000 gf * *p* = 0.037.

In the microhardness tests performed with a 100 gf load, the microhardness value obtained for sound enamel was 239.98 ± 17.52 HV. For demineralized enamel, a decrease in microhardness was observed (200.05 ± 56.12 HV) compared with sound enamel. For the samples treated with hydrogels, the following values were obtained: CS 1 day: 208.81 ± 14.24 HV; CS 7 days: 208.81 ± 14.24 HV; and CS 14 days: 122.39 ± 11.29 HV. For the samples treated with the CS–Xi hydrogel, the following values were obtained: CS–Xi 1 day: 126.96 ± 22.29 HV; CS–Xi 7 days: 134.52 ± 11.29 HV; and CS–Xi 14 days: 83.21 ± 8.72 HV. For the samples treated with the commercial paste (MI Paste^®^), a value of 245.32 ± 8.92 HV was obtained (Figure 3A). For the samples tested with a 500 gf load, sound enamel showed a microhardness of 286.18 ± 22.57 HV, while demineralized enamel showed a value of 196.81 ± 9.05 HV. The samples treated with the CS hydrogel showed the following values: CS 1 day: 209.77 ± 38.26 HV; CS 7 days: 398.31 ± 75.15 HV; and CS 14 days: 257.82 ± 12.89 HV. For the samples treated with the CS–Xi hydrogel, the following values were obtained: CS–Xi 1 day: 272.52 ± 7.18 HV; CS–Xi 7 days: 332.17 ± 7.19 HV; and CS–Xi 14 days: 382.86 ± 59.80 HV. For the samples treated with MI Paste^®^ for 14 days, the microhardness value obtained was 214.42 ± 26.92 HV (Figure 3B). Based on these values, statistically significant differences were identified between the demineralized enamel and the group treated with CS–Xi for 7 days. This result suggests that the hydrogel application at this time point may have contributed to changes associated with enamel recovery when compared with the demineralized condition. Figure 3C shows the results obtained with a 1000 gf load. The microhardness of sound enamel was 250.59 ± 30.15 HV, whereas demineralized enamel showed a value of 172.76 ± 67.06 HV. For the samples treated with the CS hydrogel, the following values were obtained: CS 1 day: 478.06 ± 135.07 HV; CS 7 days: 296.61 ± 70.14 HV; and CS 14 days: 202.43 ± 14.79 HV. For the samples treated with the CS–Xi hydrogel, the following values were obtained: CS–Xi 1 day: 211.88 ± 55.29 HV; CS–Xi 7 days: 392.40 ± 56.16 HV; and CS–Xi 14 days: 354.90 ± 19.63 HV. For the samples treated with the commercial paste (MI Paste^®^), a microhardness value of 644.90 ± 83.92 HV was obtained. These values indicate a statistically significant difference between demineralized enamel and samples treated with Mi Paste for 14 days, suggesting changes in enamel characteristics following treatment.

### 2.4. Microbiological Analysis

**Figure 4:**
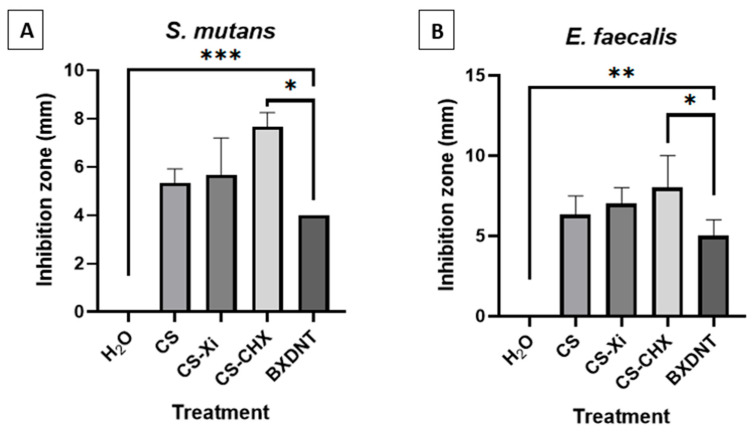
Microbiological analysis. (**A**) *S. mutans* * *p* = 0.0180, *** *p* < 0.0001; (**B**) E. faecalis * p = 0.0396, ** *p* = 0.0017.

**Figure 5:**
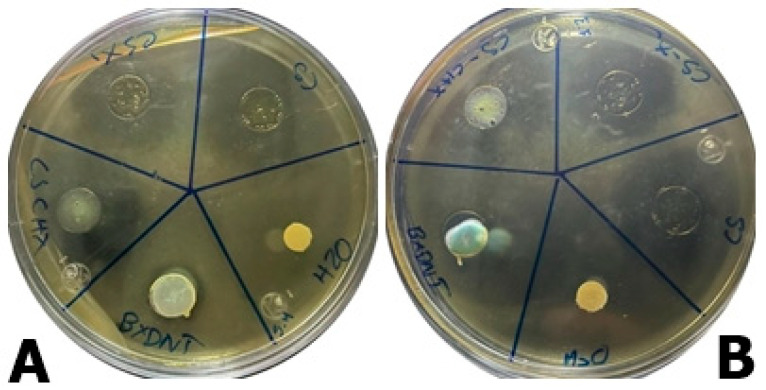
Kirby-Bauer assay. (**A**) Inhibition halos for *S. mutans*. (**B**) Inhibition halos for E. faecalis.

In the microbiological analysis against *S. mutans*, the following inhibition zones were observed. In Group 1 (control group), distilled water was used, and no inhibition zone was observed. In Group 2 (CS), an inhibition zone of 5.3 ± 0.57 mm was recorded. In Group 3 (CS-Xi), a value of 5.6 ± 1.5 mm was obtained. In group 4 (CS-CHX), an inhibition zone of 7.6 ± 0.57 mm was observed. For the commercial paste Bexident^®^, an inhibition zone of 4.0 ± 0 mm was obtained (Figure 4A and Figure 5A).

For *E. faecalis*, in Groups 1 (control group) distilled water was used in and no inhibition zone was observed. In Group 2 (CS), an inhibition zone of 6.3 ± 1.15 mm was recorded. In Group 3 (CS-Xi), a value of 7.0 ± 1.0 mm was obtained. In Group 4 (CS-CHX), an inhibition zone of 8.0 ± 2.0 mm was observed. For the commercial paste Bexident^®^, an inhibiton zone of 5.0 ± 1.0 mm was obtained (Figure 4B and Figure 5B).

### 2.5. Discussion

The main finding of this study was that the chitosan-xylitol hydrogel (CS-Xi) significantly promoted enamel remineralization, compared with the control groups.

The FTIR spectrum of the CS 4% hydrogel showed characteristic peaks corresponding to the stretching vibrations of the amino (NH) group, as well as absorption bands associated with C=O stretching, which are typical of amide groups linked to amino functionalities. These findings are consistent whit those reported by Jamnongkan and Kaewpirom [20], who developed a chitosan hydrogel, presenting vibrational bands at 1651 cm^−1^ and 1562 cm^−1^, values that are comparable to those observed in the present hydrogel formulation.

The spectrum corresponding to the chitosan (4%)-xylitol (5.4%) hydrogel showed vibrational bands in the 1421 cm^−1^, indicating torsional vibrations of OH^−^ group which are characteristic of the xylitol molecular structure [21]. Q. Previous studies have reported similar spectral characteristics for chitosan-based materials. Q. Li and colleagues [22] reported absorption bands for chitosan at approximately 1076 cm^−1^, as well as bands associated with OH^−^ groups near 3431 cm^−1^ [21]. Similarly, Musat and collaborators [7] reported absorption bands corresponding to OH- functional groups in the ranges of 1595 cm^−1^ and 3711 to 3780 cm^−1^. Additional bands observed around 1660 cm^−1^–1667 cm^−1^ correspond to C=O stretching vibrations, which are characteristic of amide-type bonds associated with chitosan [10,21]. Furthermore, bands observed near 1562 cm^−1^ correspond to torsional vibrations of the amine (NH_2_) groups, while Q. Li et al. [22] also reported amino group vibrations in the range of 1722 and 823 cm^−1^. In addition, bands are detected at 1012, 873 and 648 cm^−1^ correspond to C-H bending vibrations, which are characteristic of the molecular structure of the analyzed compounds [21]. These findings are consistent with spectral characteristics observed in the present study.

The application of the 4% chitosan hydrogel combined with 5.4% xylitol for 7 days showed a notable change in the enamel remineralization process. The corresponding SEM image revealed the formation of a regenerated surface layer, characterized by rod-like structures arranged in a similar orientation and exhibiting increased structural density, suggesting progressive enamel remineralization. These findings are consistent with those reported by Elwardani et al. [23], who observed minimal differences in remineralization between teeth treated with a remineralizing paste and those stored in natural saliva. The authors suggested that the presence of xylitol in artificial saliva contributes to remineralization due to its ability to form complexes with calcium ions, thereby enhancing mineral deposition and supporting enamel repair.

After 14 days of application, the enamel surface appeared more compact, with a considerable reduction in demineralized areas. However, the SEM images corresponding to the samples treated with CS–Xi for 14 days, stored in deionized water and saliva, respectively (Figure 2l, showed the presence of an irregular mineralized layer and partially disorganized rod-like structures. Surface coverage appeared uneven, as isolated areas of micropores and cavities were still observed across the enamel surface.

These findings are consistent with those reported by Musat and collaborators [7], who observed that a chitosan-based hydrogel combined with agarose, when applied for 7 days, promoted the formation of a remineralized layer of approximately 7 μm. However, when the application time was extended to 10 days, a decrease in enamel mineralization was reported. This phenomenon was attributed to the increased presence of amorphous apatite phases, residual chitosan phases, and the growth of poorly oriented nanocrystals, which may compromise the structural organization of the mineralized layer.

The results obtained in the present study show a similar trend, suggesting that prolonged exposure to the hydrogel may promote mineral deposition while simultaneously generating structural irregularities associated with incomplete crystal organization.

For the samples treated with MI Paste^®^, the presence of compact surface areas with moderate roughness and a high number of well-defined interprismatic spaces was observed. These findings are consistent with those reported by Cherian and collaborators [24], who stated that casein phosphopeptide (CPP) derivatives exhibit anticariogenic activity through a topical mechanism.

This effect is attributed to their ability to modulate the levels of bioavailable calcium and phosphate ions, maintaining a supersaturated ionic environment with respect to enamel minerals. Additionally, CPP-based systems have been shown to exert buffering effects on dental plaque, enhance remineralization processes, and reduce hydroxyapatite dissolution, thereby promoting enamel stability.

On the other hand, the microhardness analysis showed that the samples subjected to 100 g of load on sound enamel exhibited a microhardness of 239.98 ± 17.52 kg/mm^2^ (Table 2A). This value differs from those reported by Salinovic et al. [25], who obtained three different microhardness values for sound enamel: 366.00 ± 18.93, 343.52 ± 26.66, and 393.05 ± 16.14 kg/mm^2^, corresponding to their respective experimental groups. Similarly, Musat et al. [10] reported a microhardness value of 297.84 kg/mm^2^ for sound enamel, which is closer to the values obtained in the present studies.

Calvano and collaborators [26] reported four microhardness values, considering different gene genotypes: 245.4 ± 67.8 kg/mm^2^, 233.1 ± 68.2 kg/mm^2^, 240.5 ± 81.6 kg/mm^2^, 243.5 ± 37.2 kg/mm^2^, which are consistent with the results obtained in the present study for sound enamel.

In the analysis of the demineralized enamel samples, a microhardness value of 200.05 ± 56.12 kg/mm^2^ was obtained (Table 2A). Similarly, Salinovic et al. [25] reported values of 190.30 ± 23.71, 192.73 ± 16.37 and 201.90 ± 32.52 kg/mm^2^, which are in agreement with the results obtained in the present study. In addition, Musat and collaborators [7] reported a comparable value of 171.36 kg/mm^2^ for demineralized enamel.

Regarding the samples treated with chitosan hydrogel (CS), the present study we obtained microhardness values of 208.81 ± 14.24 kg/mm^2^ after 1 day of application, 208.81 ± 14.24 kg/mm^2^ after 7 days, and 122.39 ± 11.29 kg/mm^2^ after 14 days (Table 2A).

In contrast Pawar et al., [27] reported that demineralized enamel treated with a chitosan hydrogel for 10 days exhibited a microhardness value of 345.6 kg/mm^2^, which is considerably higher than the values obtained in the present study.

The samples that were treated with the chitosan-xylitol hydrogel (CS-Xi) and subjected to a 100 g load showed the following microhardness values: 126.96 ± 22.29 after 1 day, 134.52 ± 11.29 345.6 kg/mm^2^ after 7 days, 83.21 ± 8.72 345.6 kg/mm^2^ after 14 days (Table 2A).

In comparison, Siqueira et al. [16], reported that the application of xylitol-based varnish for 10 days resulted in a microhardness value of 191.3 ± 65.9 kg/mm^2^, demonstrating an increase in microhardness compared with the demineralized enamel control, which showed a value of 141.2 ± 55.2 kg/mm^2^ in the same study.

The lower microhardness values observed in the present study may be associated with differences in xylitol delivery systems, hydrogel formulation, and mineral availability, which may influence the degree of enamel remineralization and mechanical recovery.

The control samples treated with the commercial remineralizing paste MI paste^®^, when subjected to a 100 gf load applied for 10 s, showed a microhardness value of 245.32 ± 8.92 kg/mm^2^ (Table 2A). Similarly, Behrouzi [28] reported that demineralized enamel treated with MI paste^®^ achieved a microhardness value of 355.13 ± 34.59 kg/mm^2^, which is higher than the value obtained in the present study.

The samples subjected to a 500 gf load applied for 10 s showed that sound enamel exhibited a microhardness value of 286.18 ± 22.57 kg/mm^2^ (Table 2B). Similarly, Deswal [29] reported a microhardness value of 296.923 ± 6.108 kg/mm^2^, for sound enamel, which is consistent with the values obtained in the present study, considering the applied load (Table 2B).

Samples treated with chitosan hydrogels (CS) and subjected to a 500 gf load showed microhardness values of 209.77 ± 38.26 kg/mm^2^ after 1 day, 398.31 ± 75.15 kg/mm^2^ after 7 days, and 257.82 ± 12.89 kg/mm^2^ after 14 days (Table 2B).

Similarly, Al-Ward et al. [30] reported a microhardness value of 233.970 ± 63.623 kg/mm^2^ after applying a 500 gf load for 15 s to enamel treated with chitosan for 10 days, which is consistent with the value obtained in the present study after 14 days 257.82 ± 12.89 kg/mm^2^.

In the antimicrobial assay, the agar diffusion method (inhibition halo test) was used to evaluates antibacterial activity against *Streptococcus mutans* ATCC 25175.

The results showed an inhibition zone of 0 mm for the deionized water control, 5.3 ± 0.57 mm for the chitosan (CS) control, 5.6 ± 1.5 mm for the chitosan-xylitol group (CS-Xi), 7.6 ± 0.57 mm for the chitosan-chlorhexidine gel (CS-CHX), and 4 mm for the control group treated with the commercial chlorhexidine paste Bexident^®^ (Table 3A, Figure 5A).

Similarly, Flores-Espinoza and collaborators [31] reported that chitosan exerts antimicrobial activity by disrupting bacterial cells membranes through the displacement of Ca^2+^ ions from anionic sites, leading to increased membrane permeability and cell damage.

In the antimicrobial assay using the *Enterococcus faecalis*, the inhibition zone for the deionized water control was 0 mm, while the chitosan hydrogel (CS) showed an inhibition zone of 6.3 ± 1.15 mm, the chitosan-xylitol hydrogel (CS-Xi) 7 ± 1 mm, the chitosan-chlorhexidine hydrogel (CS-CHX) 8 ± 2 mm, and for the commercial paste Bexident^®^ 5 ± 1 mm (Table 3B) (Figure 5B). Previous studies have demonstrated that the antimicrobial activity of chitosan is mainly associated with the presence of positively charged amino groups, which interact with negatively charged components of bacterial cells walls, leading to structural damage and increased membrane permeability [31]. Xylitol, a natural sugar alcohol commonly derived from sources such as birch, exhibits anticariogenic properties due to its inability to be metabolized by most oral bacteria. Although its exact antibacterial mechanism remains unclear, it has been suggested that xylitol interferes with bacterial energy metabolism, reducing bacterial growth and acid production [32].

This study has some limitations that should be acknowledged. First, biocompatibility analyses of the hydrogel formulation were not performed, which limits the understanding of its biological safety and potential cellular interactions. Although chitosan is generally considered a biocompatible biomaterial, its biological performance may vary depending on concentration, degree of deacetylation, and formulation characteristics. Additionally, the potential allergenic risk associated with chitosan should be considered, particularly in individuals with hypersensitivity to shellfish-derived products, despite evidence suggesting that allergenic proteins are generally removed during chitosan processing. Therefore, further studies evaluating cytocompatibility, biocompatibility, and safety under oral conditions are necessary before clinical translation. Although SEM analysis enabled the assessment of surface morphological changes among the experimental groups, characteristic rod-like enamel structures were not clearly visualized under the imaging conditions employed, limiting the interpretation of enamel prism organization. Additionally, antimicrobial activity was evaluated solely using the Kirby-Bauer agar diffusion assay, which provides only a preliminary assessment and does not offer quantitative information regarding bacterial viability, biofilm inhibition, or bactericidal activity. Therefore, these findings should be interpreted with caution, and future studies incorporating higher-resolution SEM analyses, cross-sectional imaging, and complementary antimicrobial assays are recommended to provide a more comprehensive characterization of the material.

## 3. Conclusions

Samples treated with 4% chitosan hydrogel combined with 5.4% xylitol (CS-Xi) for 7 days showed an organized enamel surface morphology and changes in the Ca/P ratio, suggesting a possible remineralization effect. Longer treatment periods (>10 days) resulted in the formation of an irregular surface layer, which can negatively affect mineral deposition and microhardness. Furthermore, the CS-Xi hydrogel demonstrated antibacterial activity comparable to that of the commercial control Bexident^®^. These findings suggest that CS-Xi may represent a promising acellular approach for enamel repair with antibacterial potential, particularly after approximately 7 days of application.

## 4. Materials and Methods

### 4.1. Sample Preparation

The collection, selection, and preparation of the samples were carried out with informed consent and approval from the Ethics Committee of the Academic Unit of Dentistry at the Autonomous University of Zacatecas, under registration number (CEI-UAO/UAZA0007/2023). The sample size was established based on a modification of methodologies previously reported in similar studies. The methodological adjustment was made to allow the inclusion of a larger number of experimental groups while preserving experimental feasibility and adequate replication for reproducible results. A total of 60 samples were included in the study [33]. The samples were distributed into four groups of 15 specimens each, which were further divided into five subgroups consisting of three specimens per subgroup. The inclusion criteria were caries-free molars or premolars, while the exclusion criteria included carious, fractured, enamel-defective, or anterior teeth. The teeth were then washed three times for ten minutes each with deionized water in an ultrasonic cleaner. The cleaned samples were sectioned from mesial to distal using a low-speed diamond disc under continuous water cooling. The resulting sections (1.5–2 mm wide) were demineralized in 37% phosphoric acid for 30 s. Afterward, they were placed in an ultrasonic bath with deionized water to remove any residual acid. The samples were dried and stored at 4 °C until use.

### 4.2. Preparation of the Hydrogels

For the preparation of the reagents, chitosan powder (degree of deacetylation ≥ 75% and high molecular weight), xylitol (purity ≥ 99% and molecular weight of 152.15 g/mol) from Sigma-Aldrich^®^ (St. Louis, MO, USA), and acetic acid (99.7% purity) from Golden Bell^®^ (Golden Bell Biological Technology Co., Ltd., Beijing, China) were used. Three types of hydrogels were created from the obtained reagents; for the 4% chitosan hydrogel (CS 4%), 9 mL of deionized water were used, to which 0.4 mL of acetic acid, 0.4 g of chitosan, and 0.04 mL of glycerol (analytical grade, Sigma-Aldrich^®^, St. Louis, MO, USA) were added. The mixture was then manually stirred at 37 °C until the desired consistency was achieved. For the 5.4% chitosan-xylitol hydrogel (CS-Xi 5.4%), the same procedure was followed, with the addition of 0.54 g of xylitol. The mixture was then manually stirred at 37 °C until the desired consistency was achieved. For the chitosan-chlorhexidine hydrogel, the same procedure was followed as for the 4% chitosan hydrogel, with the addition of 0.2% chlorhexidine. The mixture was then manually stirred at 37 °C until the desired consistency was obtained. CS-based hydrogel and CS-Xi-based hydrogel were used in the remineralization protocol, whereas CS-CHX was employed in the antibacterial protocol.

### 4.3. Remineralization Treatment

For the remineralization treatment, the (CS) and (CS-Xi) hydrogels were applied to the demineralized samples and stored in deionized water or artificial saliva [17] for 1, 7, and 14 days. For the control group, the commercial toothpaste MI paste^®^ (GC corporation, Tokyo, Japan) applied using the same protocol, for 1, 7, and 14 days. At the end of the treatment period, the samples were removed, rinsed with deionized water, and air-dried.

### 4.4. Preparation of Artificial Saliva

Artificial saliva was prepared according to the protocol described by Engelhart, Popescu, and Bernhardt [34], in which 4200 mg/L of NaHCO_3_, 500 mg/L of NaCl and 200 mg/L of KCl were used. These reagents were dissolved in distilled water, and the pH was adjusted to 7.3.

### 4.5. Structural Analysis

Fourier transform infrared (FTIR) spectra of the hydrogels were recorded in transmittance mode over the 4000–400 cm^−1^ range using an IRAffinity-1S spectrophotometer (Shimadzu Corporation, Kyoto, Japan). Aliquots of 1 mL from each hydrogel formulation were incubated at 60 °C for 48 h to obtain a dry film. The resulting films were then stored in vacuum-sealed bags for transport to the Bionanomaterials Laboratory at the Universidad Autónoma de San Luis Potosí (UASLP). Subsequently, the samples were mounted on the sample holder and analyzed using Fourier-transform infrared spectroscopy (FTIR).

### 4.6. Morphological and Compositional Analysis

For the structural analysis, cross-sectional views of the sound, demineralized, and hydrogel-treated samples was used, the specimens were dehydrated using graded isopropyl alcohol solutions at 25%, 40%, 50%, 75%, 80%, 90%, and 100% concentrations. The samples were immersed for 30 min in each concentration. After dehydration, the samples were incubated at 37 °C overnight. Subsequently, the samples were gold-sputter coated prior to analysis. Scanning electron microscopy coupled with energy-dispersive X-ray spectroscopy (SEM-EDX) was performed using a JEOL, JSM-6510 scanning electron microscope (JEOL Ltd., Tokyo, Japan).

### 4.7. Mechanical Analysis

Mechanical analysis of cross-sectioned, sound, and hydrogel-treated samples was performed using Vickers microhardness testing (Vickers hardness test according to ISO 6507/ASTM E384-17) [35,36] with loads of 100, 500, and 1000 gf applied for 10 s using a Micromet II microhardness tester (Buehler Ltd., Lake Bluff, IL, USA). Three indentations were performed on each sample, spaced 4 μm apart. Microhardness values were calculated in accordance with the ASTM E384-17 standard test method.

### 4.8. Microbiological Analysis

Microbiological analysis was performed using the Kirby-Bauer disk diffusion method with two bacterial strains: *S. mutans* (ATCC 25175) and *E. faecalis* (ATCC 29212). The bacterial strains were inoculated into tryptic soy broth (TSB) (BD Bioxon^®^, Mexico City, Mexico) and incubated for 24 h at 37.5 °C. After incubation, the bacterial cultures were streaked onto tryptic soy agar (TSA) (BD Bioxon^®^, Mexico City, Mexico) using a sterile inoculation loop and incubated again for 24 h at 37.5 °C. For preparation of the McFarland standard suspension, bacterial colonies were transferred into sterile distilled water and adjusted using a spectrophotometer at a wavelength of 625 nm until an absorbance of 0.08–0.12 was obtained, corresponding to a bacterial concentration of approximately 1.5 × 10^8^ CFU/mL (0.5 McFarland standard).

After achieving the desired concentration, five wells, approximately 10 mm in diameter, were prepared in the agar plates, and the hydrogels under study were added as follows: deionized water was added to well 1 (negative control), 4% chitosan hydrogel to well 2, 4% chitosan-5.4% xylitol hydrogel to well 3, 4% chitosan-2% chlorhexidine hydrogel to well 4 (positive control), and Bexident^®^ commercial paste (ISDIN, Madrid, Spain) to well 5. The plates were then incubated at 37.5 °C for 24 h.

### 4.9. Statistical Analysis

Statistical analysis was performed based on a k-sample design. Normality was evaluated using the Shapiro–Wilk test, confirming parametric distribution of the data. Therefore, a one-way ANOVA followed by Tukey’s post hoc test was applied for multiple comparisons. Statistical significance was set at *p* < 0.05. Data analysis was performed using GraphPad Prism version 9.0.2 (GraphPad Software, San Diego, CA, USA).

## Figures and Tables

**Table 1:** EDX analysis.

Sample	Ca/P Ratio
Sound enamel	2.6%
Demineralized enamel	2.7%
1 day	1.9%
7 days	1.8%
14 days	2.6%

**Table 2:** Microhardness analysis. (**A**) 100 gf; (**B**) 500 gf; (**C**) 1000 gf.

	(A)	(B)	(C)
Microhardness Group	(100 gf HV, kg/mm^2^) Mean ± SD	(500 gf, kg/mm^2^) Mean ± SD	(1000 gf, kg/mm^2^) Mean ± SD
Sound enamel	239.98 ± 17.52	286.18 ± 22.57	250.59 ± 30.15
Demineralized enamel	200.05 ± 56.12	196.81 ± 9.05	172.76 ± 67.06
CS 1 day	208.81 ± 14.24	209.77 ± 38.26	478.06 ± 135.07
CS-Xi 1 day	126.96 ± 22.29	272.52 ± 7.18	211.88 ± 55.29
CS 7 days	208.81 ± 14.24	398.31 ± 75.15	296.61 ± 70.14
CS-Xi 7 days	134.52 ± 11.29	332.17 ± 7.19	392.4 ± 56.16
CS 14 days	122.39 ± 11.29	257.82 ± 12.89	202.43 ± 14.79
CS-Xi 14 days	83.21 ± 8.72	382.86 ± 59.80	354.90 ± 19.63
MP 14 days	245.32 ± 8.92	214.42 ± 26.92	644.90 ± 83.92

**Table 3:** Microbiological analysis. (**A**) *S. mutans* (**B**) *E. faecalis*.

**(A)**				
***S. mutans***	**Plate 1 (mm)**	**Plate 2 (mm)**	**Plate 3 (mm)**	**Mean ± SD (mm)**
Group 1 (H_2_O)	0	0	0	0
Group 2 (CS)	5	6	5	5.3 ± 0.57
Group 3 (CS-Xi)	7	6	4	5.6 ± 1.5
Group 4 (CS-CHX)	8	8	7	7.6 ± 0.57
Group 5 (BXDNT)	4	4	4	4 ± 0
**(B)**				
***E. faecalis***	**Plate 1 (mm)**	**Plate 2 (mm)**	**Plate 3 (mm)**	**Mean ± SD (mm)**
Group 1 (H_2_O)	0	0	0	0
Group 2 (CS)	7	7	5	6.3 ± 1.15
Group 3 (CS-Xi)	8	7	6	7 ± 1
Group 4 (CS-CHX)	10	8	6	8 ± 2
Group 5 (BXDNT)	4	6	5	5 ± 1

## Data Availability

The original contributions presented in this study are included in the article. Further inquiries can be directed to the corresponding author.

## Abbreviations

The following abbreviations are used in this manuscript:

CS	Chitosan
CS-Xi	Chitosan-Xylitol
CS-CHX	Chitosan-Chlorhexidin
MP	MI Paste^®^
